# MRI Analysis of the Wrist: Does the Presence of Palmaris Longus Affect Median Nerve Position?

**DOI:** 10.1177/15589447251314145

**Published:** 2025-01-30

**Authors:** Victor B. Chavez, Dean W. Smith

**Affiliations:** 1 The University of Texas Health Science Center at Houston, USA

**Keywords:** median nerve, MRI, orthopedic surgery, palmaris longus, wrist

## Abstract

**Background:**

Understanding the median nerve’s position relative to surrounding anatomy is essential; however, there are many variations among individuals. This study assesses differences in median nerve position with or without palmaris longus (PL). We hypothesize that PL presence alters median nerve position, resulting in a greater distance to the skin volar surface, a decreased distance to the radius volar surface, and an increased distance to the flexor carpi radialis (FCR).

**Methods:**

1193 wrist magnetic resonance imaging (MRI) studies were retrospectively reviewed from 2019 to 2023. One hundred adults ages 18 to 50 meeting criteria were included for a power > 80%: 50 wrist axial plane MRIs (distal radial-ulnar joint level) with PL and 50 without PL. Measurements included the distance from the median nerve center to the skin volar surface, radius volar surface, and FCR center. Statistical analysis included Fisher exact tests and Mann-Whitney U Test (median, ranges), with significance at *P*-value < 0.05.

**Results:**

Individuals with PL had a greater distance between the median nerve and skin volar surface. The presence of PL exhibited no discernable difference in the distance between the median nerve to the radius volar surface or the FCR center. Palmaris longus presence or absence did not affect the radial/ulnar positioning of the median nerve to the FCR center.

**Conclusions:**

PL presence results in a deeper median nerve position within the wrist in relation to the skin volar surface. This knowledge is crucial for musculoskeletal specialists, especially during volar approach wrist surgeries and when administering anesthetic or therapeutic injections to the median nerve.

## Introduction

The median nerve plays a vital role in the motor and sensory functional capabilities of the hand. Individual variations in hand and wrist skeletal, tendinous, muscular, arterial, and neurologic anatomy are not uncommon and have been frequently reported in the literature.^1^ Prior studies have also suggested that median nerve position can be altered by disease states and wrist position.^2,3^ Volar wrist procedures such as open or endoscopic carpal tunnel surgery, distal radius fracture management, and the administration of median nerve anesthetic blocks or therapeutic injections, require an understanding of the median nerve location, as well as variations in individual anatomy.

Many extensor and flexor tendons reside outside of the carpal tunnel. This study focuses on the relationship between 2 tendons: the palmaris longus (PL) and flexor carpi radialis (FCR) and their anatomical position with the median nerve. The PL originates from the medial epicondyle of the humerus and passes along the superficial compartment of the forearm, inserting into the palmar aponeurosis and is innervated by the median nerve.^4^ Although secondary to the FCR and FCU, the PL when present contributes to active wrist flexion.^5^ The PL is also an important tendon used in reconstructive hand surgery and often used as a reference landmark for locating the median nerve in the distal forearm. Although uncommon with open and endoscopic carpal tunnel release, iatrogenic injury to nerves, tendons, and vessels has been reported.^6^ Other reports revealed that a surgeon can mistake the median nerve for PL.^7-9^ Misidentification of PL for the median nerve may be due to surgical confusion, limited visibility, time constraints, and/or absent or atypical muscle anatomy.^8^ A 2015 literature review reported that the absence of PL within individuals can range from 1.5% to 63.9%.^10^ However, it is unknown whether variations, such as the absence of a PL, may affect median nerve anatomical location and depth. Little evidence exists regarding the effects of this anatomical variation on the positioning of the median nerve.

This study aims to determine whether there is a difference in the anatomical position of the median nerve in neutral wrist position, with or without a PL, through a retrospective magnetic resonance imaging (MRI) review. We hypothesize that the presence of the PL will result in the median nerve having a greater distance to the skin volar surface, a decreased distance between the median nerve to the volar radial surface, and an increased distance between the median nerve and FCR, resulting in a significant position difference of the median nerve between patients with and without a PL. This knowledge is important to the musculoskeletal surgeon in understanding individual differences in median nerve position during surgical exposure and therapeutic injections.

## Materials and Methods

### Study Design

Institutional review board approval was obtained for the retrospective imaging study. The images were viewed on high-definition monitors and measurements were conducted using Synapse PACS (Fujifilm Healthcare) and Centricity (GE Healthcare) Imaging software.

### Study Population

A study population of 1193 outpatient and inpatient wrist MRIs between 2019 and 2023 was selected from the hospital imaging database. The specific population of interest was set between the ages of 18 and 50 to mitigate any potential confounding factors related to age, such as arthritis or other degenerative conditions that could compromise the integrity of the wrist joint and potentially influence positioning of the flexor tendons or median nerve.

Magnetic resonance imaging studies with mass effects, such as volar ganglion cysts, tumors, or inflammation, were excluded from the final cohort as this could distort the local anatomy and displace or compress the surrounding tissues. Bone abnormalities, such as fractures and dislocations, were also excluded as they could alter the spatial relationships between the muscles, nerves, and bones of the forearm or carpus. Finally, MRI artifacts caused by metallic implants, motion, and technical errors were excluded as they distorted the quality of the MR images, thus making it difficult to accurately assess the wrist anatomy.

**Figure 1.**
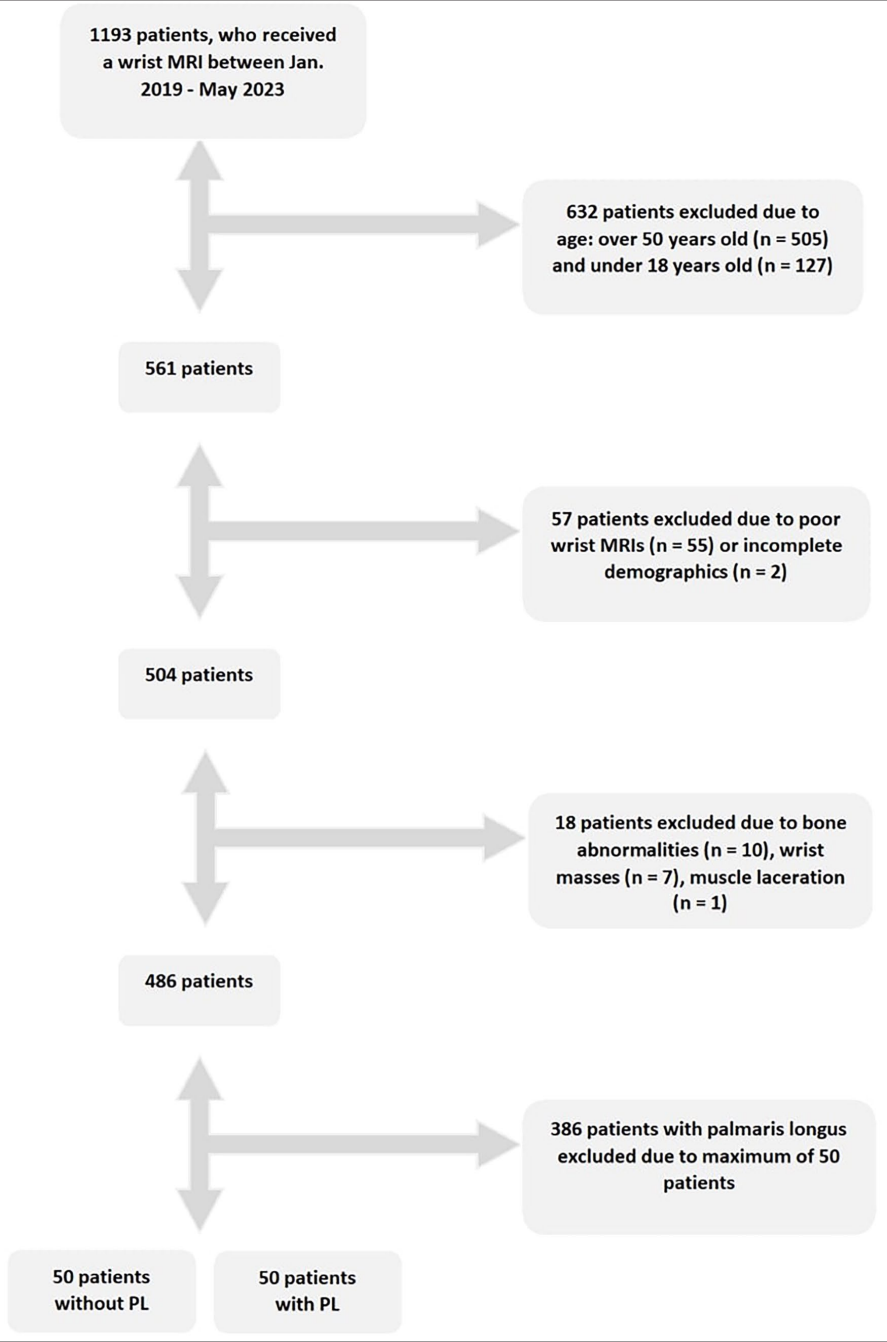
Flow diagram of study participants. Figure 1 shows graphic representation of how participants were selected based on the elimination criteria. *Note*. MRI = magnetic resonance imaging; PL = palmaris longus.

A total study sample size of 100 individuals was established to achieve a power of 80% or greater. To avoid duplicate records, only the primary recorded MRI was included for analysis. Figure 1 summarizes the steps involved in screening the patients who met the criteria for inclusion. Using hospital imaging databases, 1193 individuals from 2019 to 2023 were pooled in this study using the following search terms: wrist MRI, 2019, 2020, 2021, 2022, and 2023. Records were screened for those aged between 18 and 50, which narrowed the pool to 561. The MRIs were then individually analyzed for any artifacts or incomplete demographics, such as no recorded age or no MRI tied to their medical report, which reduced the pool to 504. Further screening for any mass effect, bone, tendon, or muscle abnormalities narrowed the pool to 486. Finally, once 50 patients with a PL were recorded, 386 patients were removed from the study as maximum study enrollment was reached for the PL group. Through this elimination process, 2 groups of 50 individuals with and without a PL remained.

### Study Protocol

**Figure 2.**
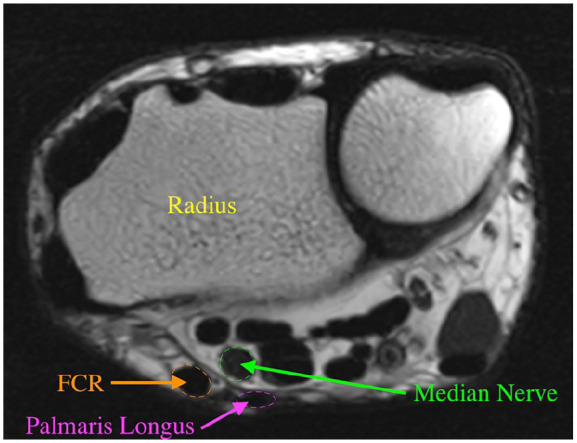
Wrist anatomy (DRUJ level). Figure 2 illustrates a magnetic resonance image of the anatomical structures of the wrist at the DRUJ level, which were utilized in the study. *Note.* DRUJ = distal radial-ulnar joint; FCR = flexor carpi radialis.

**Figure 3.**
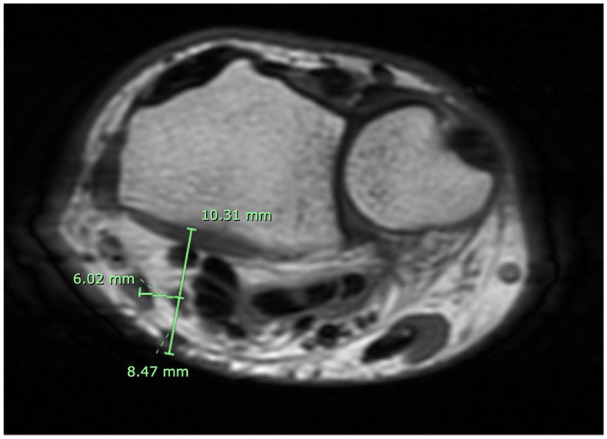
With palmaris longus. *Note.* Figure 3 illustrates a magnetic resonance image of the 3 measurements recorded for one of the 50 wrists with a palmaris longus. Distance of 8.47 mm from the center point of the median nerve to the volar skin surface. Distance of 6.02 mm between the center point of the median nerve to the center point of the flexor carpi radialis. Distance of 10.31 mm between the center point of the median nerve to the radius volar surface.

**Figure 4.**
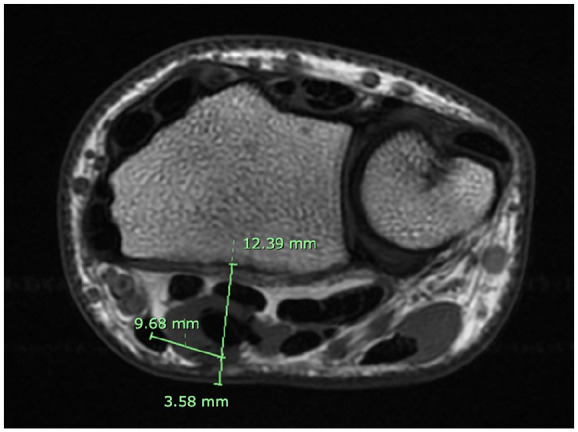
Without palmaris longus. *Note.* Figure 4 illustrates a magnetic resonance image of the 3 measurements recorded for one of the 50 wrists without a palmaris longus. Distance of 3.58 mm from the center point of the median nerve to the volar skin surface. Distance of 9.68 mm between the center point of the median nerve to the center point of the flexor carpi radialis. Distance of 12.39 mm between the center point of the median nerve to the radius volar surface.

The first 100 individuals with and without a PL within the 18 to 50 age range, who met the inclusion criteria and exhibited no signs of mass effect, bone abnormalities, or MRI artifacts were selected and de-identified. Data was obtained for each static MRI at the distal radial-ulnar joint (DRUJ) level (Figure 2) using the axial plane image sequences. One examiner collected 3 measurements on the images with and without PL: (1) distance from the center point of the median nerve to the volar skin surface; (2) distance between the center point of the median nerve and the center point of the FCR; and (3) distance between the center point of the median nerve to the radius volar surface. These measurements reflect the depth of the median nerve at the level of the DRUJ and its position in relation to the FCR tendon. Figures 3 and 4 show measurements of wrists with and without PL at the DRUJ level. From this data, we analyzed the anatomical importance of the presence and absence of the PL in relation to the position of the median nerve.

### Statistical Analysis

Sample size was determined by power analysis to achieve a power greater than or equal to 80%. Power designation allow the 2 groups to detect a difference with a significance of 95%, indicating statistical significance as less than .05. Fisher exact tests were used for the following categorical variables: right or left wrist, sex, and PL presence or absence. The Mann-Whitney U test was used to examine the continuous variables including age and the measured distances between the median nerve to skin volar surface, radius volar surface, and FCR due to a nonnormal distribution, thus, median and range are reported. All analyses were performed with an alpha level of .05.

## Results

### Data Set Overview

One thousand one hundred and ninety-three wrist MRIs were reviewed and recorded over a 5-year period (2019-2023). From this data set, the first 100 wrist images with and without a PL that met the inclusion criteria were analyzed. Once 50 wrist images with a PL were identified, the remaining eligible PL images were excluded. The 50 individuals with a PL and 50 individuals with an absent PL were assessed.

### Median Nerve to Skin Volar Surface Distance Comparison

**Figure 5.**
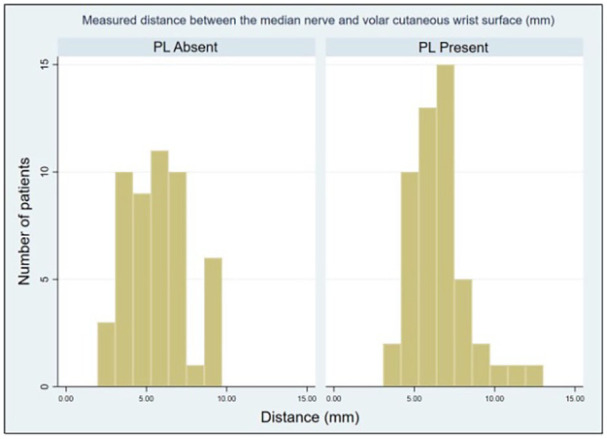
Histogram of significant results. Figure 5 illustrates the range of measured distances between the median nerve to the skin volar surface with and without palmaris longus (PL).

When the 2 groups of 50 individuals with and without a PL were compared, we found that the distance difference between the median nerve to the skin volar surface was statistically significant (*P* < .05) as shown in Table 1. The distance for patients with PL (median = 6.4, ranging from 3.8 to 13) was significantly greater than for wrists without PL (median = 5.5, ranging from 2 to 9.5). Figure 5 illustrates the range of measurement differences between the median nerve and skin volar surface, comparing cases with and without the presence of PL.

**Table 1.** Results.

Variable	Palmaris present (n = 50)	Palmaris absent (n = 50)	*P*-value
Wrist side			.42
Right	31 (62%)	27 (54%)	
Left	19 (38%)	23 (46%)	
Sex			.23
Male	26 (52%)	20 (40%)	
Female	24 (48%)	30 (60%)	
Median age (Range, Years)	32.5 (18 to 50)	38.5 (18 to 50)	**.04**
Mean distance between median nerve and:			
Skin volar surface (mm)	6.4 (3.8 to 13)	5.5 (2 to 9.5)	**.01**
Radius volar surface (mm)	10.8 (6.9 to 15.5)	11.2 (5.1 to 16.4)	.82
Flexor carpi radialis (mm)	6.0 (3.1 to 9.1)	6.3 (3 to 11)	.14

*Note*. *P*-value: Probability value of statistical significance. Bolded values: Statistically significant results (*P* < .05). mm = millimeters.

### Median Nerve to Radius Volar Surface Distance Comparison

The distance for patients with PL (median = 10.8, ranging from 6.9 to 15.5) showed no difference in the median nerve to the radius volar surface compared to the group without PL (median = 11.2, ranging from 5.1 to 16.4). This was not statistically significant.

### Median Nerve to FCR Center Distance Comparison

The distance for patients with PL (median = 6, ranging from 3.1 to 9.1) showed no difference in the median nerve to the FCR center compared to the group without PL (median = 6.3, ranging from 3 to 11). This was not statistically significant.

### Demographic Factors

Table 1 compares the patient demographics and their median and ranges of their measured distances of their anatomical wrist structures, with and without a PL. The results revealed that wrist side and sex were insignificant (*P* > .05) for difference of presence or absence of PL. These findings demonstrate that PL variation was not dependent on right or left wrist or an individual’s sex in this cohort. The 2 groups with and without PL were found to differ in age and this difference was significant (*P* < .05). However, PL absence is an anatomical anomaly that is independent of age. The age difference between the 2 groups was most likely due to chance.

## Discussion

The PL is one notable structure that contributes to the anatomical positioning of the median nerve. At the neutral wrist position and DRUJ level, our study found that the presence of the PL significantly increased the distance between the volar wrist skin layer to the median nerve. Moreover, with the presence of PL, the median nerve was found to be anatomically deeper in orientation as compared to wrists without PL. These findings were as expected in our hypothesis. In contrast, when PL was absent, the median nerve was found to be more superficial, likely due to the absence of this additional muscular and tendon layer provided by the PL in the forearm. These results support that PL presence can lead to changes in anatomical variability.

Although the spatial relationship and measured distances between the median nerve to the radius volar surface and the median nerve to the center of FCR were not statistically significant, patients with PL tended to exhibit a lesser distance between the median nerve to the center of FCR (median = 6, ranging from 3.1 to 9.1). Median nerve to the volar radial surface measurements showed no discernable difference when comparing the presence (median = 10.8, ranging from 6.9 to 15.5) or absence (median = 11.2, ranging from 5.1 to 16.4) of PL. Consequently, we cannot reject the null hypothesis, as only the presence of PL resulted in the median nerve being situated at a greater distance from the skin volar surface.

Our findings have significant implications for musculoskeletal surgery. Knowledge of a more superficial median nerve, when the PL tendon is absent, is important in guiding surgical approaches and preventing iatrogenic injury. Moreover, identifying patients without a PL prior to surgery can reduce the risk of misidentification of the median nerve for the tendon during an operation. Beyond surgical implications, our findings are relevant for injection application and pain management. Awareness of a more superficial median nerve, when the PL tendon is absent, can assist clinicians during anesthetic administration and corticosteroid injections in the region.

Other studies have investigated the position of the median nerve in relation to the PL tendon. In a retrospective MR study, Hou et al^11^ observed the radial and ulnar orientation of the median nerve when PL was present. Cases without PL were not analyzed. Their study found that the median nerve was positioned more toward the midline of the wrist when PL was present, varying widely among all patients, and that the position of the median nerve was affected in patients with carpal tunnel syndrome.

In our study, the distance between the median nerve to FCR was not statistically significant between groups with and without a PL, but, unlike Hou et al, the distance between the FCR tendon and median nerve indicated a lesser distance when PL was present. Finally, our study measured the depth of the median nerve and radial/ulnar positioning in reference to the FCR tendon with and without PL, while Hou’s study only investigated the radial/ulnar orientation of the median nerve when PL was present.

In a cadaveric study, Brooks et al^12^ investigated anatomical landmarks with ultrasound to define a safe carpal tunnel injection site. Using only 7 specimens, the authors measured the anatomical relationship of the median nerve to the PL (if present) and FCR. Results revealed that on average the median nerve was 3 and 9.57 mm positioned ulnarly to the PL tendon and FCR, respectively. The authors concluded that when PL is present, the median nerve is more ulnar in position and that injections should be inserted ulnar to the FCR tendon to reduce risk of median nerve injury.

When comparing subjects with and without PL, we found no statistical difference in the distance between the FCR and median nerve at the level of the DRUJ. The differences in measurements of the median nerve to the FCR observed between Brooks’ observational study and our study can be attributed to the small sample size of 7 cadavers compared to our larger population sample of 100 live subjects.

Limitations with our study include variability of slicing position of axial MRIs by different imaging centers, leading to inconsistent slice thickness and spacing when capturing the wrist at the DRUJ level. This was minimized by recording the MRI slice closest to the DRUJ level. In addition, our study may have been influenced by nonstandardized imaging protocols and magnetic field strength. This was minimized by excluding MRIs with any noise or poor imaging quality. Also, our exclusion criteria did not consider underlying patient diagnosis or reasons for imaging. Another potential limitation was that body mass index was not collected. Body mass index could have influenced wrist size and distance between anatomical structures. We ensured that all patients were aged 18 to 50 and free of mass effect, bone abnormalities, or MRI artifacts and aimed to establish specific exclusion criteria that controlled for confounding factors from the obtained MRIs.

Our study has many merits which include a large volume of diverse patients, a thorough elimination and inclusion criteria, reproducible findings through our measurement protocol, and a powered comparison between 2 groups. These findings demonstrate that the presence and absence of PL can affect the anatomical position of the median nerve. Such insights provide musculoskeletal specialists with pre-procedural knowledge in cases involving injections or incisions near the median nerve. Surgeons and clinicians must know about anatomical variations and be able to detect a PL tendon as it can affect the location of the median nerve. Furthermore, our investigation adds to existing literature by demonstrating individual variations in wrist anatomy and the position of the median nerve, both with and without the presence of PL. Surgeons and clinicians can approach these cases knowing that the median nerve will be more superficial when the PL is absent than in patients with a PL. Application of these steps could serve to decrease iatrogenic injury to the median nerve during surgery or therapeutic injections.
